# Elimination of Oxygen Interference in the Electrochemical Detection of Monochloramine, Using *In Situ* pH Control at Interdigitated Electrodes

**DOI:** 10.1021/acssensors.0c02264

**Published:** 2021-02-22

**Authors:** Ian Seymour, Benjamin O’Sullivan, Pierre Lovera, James F. Rohan, Alan O’Riordan

**Affiliations:** Tyndall National Institute, Cork T12 R5CP, Ireland

**Keywords:** chlorine sensing, water quality monitoring, in situ pH control, amperometric sensor, interdigitated electrode array, hypochlorous acid

## Abstract

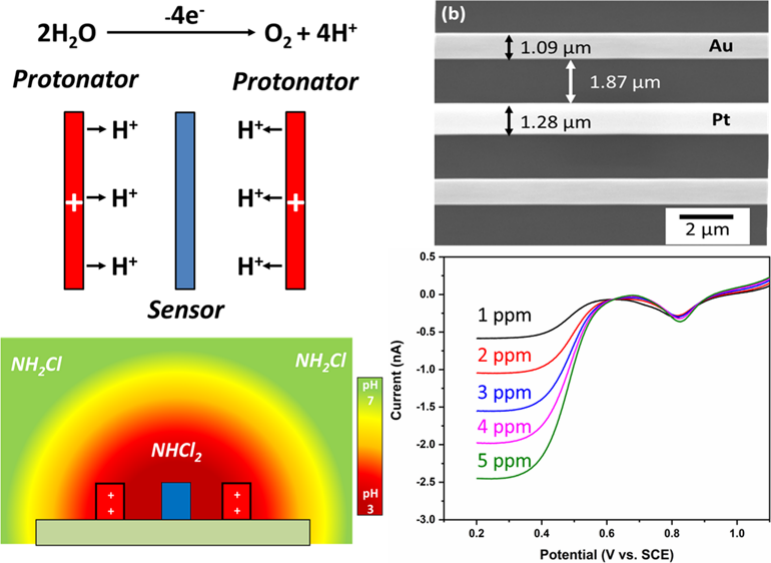

Disinfection of water systems by chloramination is a method frequently used in North America as an alternative to chlorination. In such a case, monochloramine is used as the primary chlorine source for disinfection. Regular monitoring of the residual concentrations of this species is essential to ensure adequate disinfection. An amperometric sensor for monochloramine would provide fast, reagent-free analysis; however, the presence of dissolved oxygen in water complicates sensor development. In this work, we used in-situ pH control as a method to eliminate oxygen interference by conversion of monochloramine to dichloramine. Unlike monochloramine, the electrochemical reduction of dichloramine occurs outside the oxygen reduction potential window and is therefore not affected by the oxygen concentration. Potential sweep methods were used to investigate the conversion of monochloramine to dichloramine at pH 3. The pH control method was used to calibrate monochloramine concentrations between 1 and 10 ppm, with a detection limit of 0.03 ppm. Tests were carried out in high alkalinity samples, wherein it was found that the sensitivity of this method effectively remained unchanged. Monochloramine was also quantified in the presence of common interferents (copper, phosphate, and iron) which also had no significant impact on the analysis.

Disinfection of water systems is crucial to ensure the safety of potable water and, typically, hypochlorous acid (HOCl) is used as a disinfecting agent.^1,2^ The measurement of residual chlorine is important as it can determine the progress of the disinfection process. However, it is also crucial to monitor the byproducts of the disinfection process, one of which is monochloramine (MCA). MCA is formed when HOCl reacts with ammonia (NH_3_), as shown in eq 1(3,4)
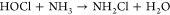
1

The formation of MCA is governed by the concentration of nitrogen relative to chlorine. This is defined as the chlorine to nitrogen ratio (Cl_2_/N). MCA is produced when the Cl_2_/N is between 3:1 and 5:1.^5,6^ For a chlorine disinfection system, formation of MCA can be undesirable as it results in the conversion of “free” chlorine to a combined chlorine species. This results in lower residual chlorine and therefore less adequate disinfection. Accurate monitoring of MCA formation in water systems reduces the risk of an improper disinfection process.

However, MCA may be formed and present in a water system not just as a byproduct of disinfection. In fact, some water system utility companies, particularly in North America, are switching to MCA as their primary water disinfectant source.^7^ While typically MCA is regarded as a weaker disinfectant, there are numerous benefits to its use in water systems. It has been shown that MCA hydrolyzes at a much slower rate than hypochlorous acid and is far more stable in UV light.^8,9^ This means that the residual chlorine is more persistent and thus a more widespread and longer lasting disinfection is achieved. It has also been found to penetrate biofilms better, resulting in superior biocidal activity.^10^ Perhaps a more significant advantage is that MCA results in the formation of less toxic byproducts. Water that has been treated with hypochlorous acid can result in the formation of trihalomethanes (THM)^11,12^ which reportedly have mutagenic, cytotoxic, and genotoxic effects, and minimization of their prevalence is desirable.^13^ MCA-treated water has been shown to have significantly lower concentrations of resulting THMs.^14^ Treatment with MCA does have its disadvantages, most typically the formation of dichloramine (DCA) and trichloramine (TCA), which form as the chlorine content increases, or the sample pH becomes acidic. The effect of sample pH on the formation of DCA and TCA is shown in the Supporting Information (SF1). Both are less powerful disinfectants than MCA and can lead to unpleasant taste and smell in the water system. More significantly, the subsequent breakdown of TCA can lead to the formation of nitrates and nitrites. High concentrations of these can lead to nitrification of the water system which is hazardous to aquatic life.^15−17^ Low concentrations result in poor disinfection, but can also be an indicator of high concentrations of organic matter in the system.^18^ Because of this, MCA levels need to be monitored in water systems. The typical concentration expected in a chloraminated system ranges from 0.6 to 5 mg/L^19^ and requires routine analysis to ensure it stays within this range. Therefore, the measurement of MCA is crucial in both chlorinated and chloraminated water systems.

Many methods exist to determine MCA concentrations, such as spectrophotometry, chemical titrations, gas chromatography, liquid chromatography, and mass spectrometry.^20−22^ A simple alternative to a lab-based approach is to use a commercially available colorimetric test kit specific for MCA.^23^ In the test, a reagent containing phenate is added to the MCA solution where it reacts to form an indophenol, which is green in colour.^24^ The issue with these methods, however, is that a skilled operator is required to do the analysis, and additional reagents are required during testing that are environmentally undesirable. They also require sampling of the water system by an operator to remove an adequate volume for measurement. This increases the time and the cost associated with carrying out a measurement. By contrast, electrochemical methods are portable, of low cost, and are highly sensitive.^25−27^ Quantification using an electrochemical method would simplify the analysis with no additional reagents required, and such devices can be deployed and accessed remotely.^28^

The electrochemical analysis of MCA is undertaken by measuring the current associated with the reduction of MCA to ammonium and chloride. This is a one-step, two electron reduction as shown in equation^20,29^
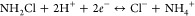
2

A key innovation in this research study is the elimination of oxygen as an interfering species. One of the major issues with electrochemical quantification of MCA is that dissolved oxygen in the solution is an interferent.^20,30,31^ Dissolved oxygen is ubiquitous, varies in concentration depending on a number of factors including temperature and altitude, and undergoes reduction in the same electrochemical window which is used for the analysis of MCA, typically between −0.5 and 0.1 V.^29,32^ For these reasons, a direct detection of MCA would also require the determination of oxygen concentration. The reduction of DCA, however, occurs at potentials completely outside of the oxygen reduction potential window (between 0.2 and 0.6 V), the reaction for which is shown in eq 3(29)
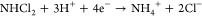
3

Conversion of MCA to DCA requires a pH shift to acidic conditions, resulting in the formation of a protonated MCA species, which breaks down to form DCA, as shown in eqs 4 and 5(33,34)
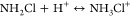
4
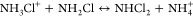
5

The conversion of MCA to DCA can be achieved using, *in situ* pH control. With this method, a protonator electrode changes the pH in the vicinity of the sensor electrode, such that the local MCA is converted to DCA. The DCA concentration is then measured by the sensor electrode *via* a reduction reaction. We have recently shown that an *in situ* pH control method can be facilitated at interdigitated microelectrodes for the detection of hypochlorous acid by protonation of the hypochlorite ion, wherein the gold oxide reduction peak was used as an indicator of the local pH.^35^ Conversion to a single species removed the complexity involved in the calibration process. Using microfabricated on-chip interdigitated arrays, the pH can be tailored to the required value by control of the applied current density.^36^ The electrochemical measurements in this work utilized a generator–collector type device composed of two combs of interdigitated electrode (IDE) arrays. A comb here refers to one-half of the interdigitated electrode array. The working electrodes are spaced 2 μm apart, while the counter electrode is 1.1 mm away from the sensors. By imposing an appropriate potential at one (“protonator”) comb of electrodes, a pH change occurs in the local environment that tailors the pH at the other (“sensor”) comb. This sensor can thus be used to perform sensing in pH conditions that differ considerably from the bulk solution. It is essential that the counter electrode is spatially well removed from the interdigitated combs to ensure that the reciprocal consumption of protons does not occur too close to the sensing electrode, which would inevitably prevent the *in situ* pH control. Using this approach, a local environment is created that is more acidic (or basic) than the bulk conditions. We apply this method for the sensing of MCA, by electrochemically shifting the pH at a sensor electrode to more acidic conditions. Converting the MCA to DCA shifts the reaction of interest outside the oxygen reduction potential window, thus removing oxygen as an interfering species and simplifying the analysis. The close spacing of the interdigitated electrodes ensures that *in situ* pH control is established by the rapid diffusion of protons, so additional convection or fluidic forces are not required. Thus, this approach has the potential for in-line analysis deployment as required, for example, in water distribution systems. The schematic in Figure 1 shows how *in situ* pH control creates a local acidic environment allowing for the sensor electrode to amperometrically detect DCA rather than MCA.

**Figure 1:**
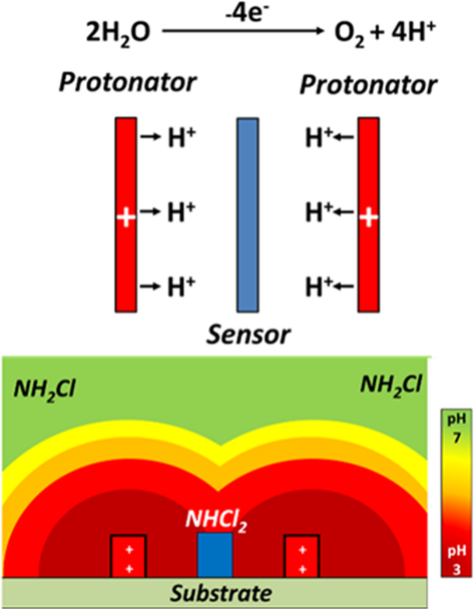
Schematic representation of the pH control method. The blue rectangle represents the sensing electrode, while the red rectangle represents the protonator.

## Experimental Section

### Electrode Fabrication

Silicon chip-based devices were fabricated using methods similar to those described by Dawson *et al.*(37−39) Each chip consisted of two combs of gold-working IDEs. A platinum pseudo reference and gold counter electrode were also employed on-chip. In brief, chips were designed to interface with external electronics via a microSD port to facilitate facile electrical connection. All of the devices were fabricated on 4 inch silicon wafers bearing a thermally grown 300 nm silicon dioxide layer. Blanket metal evaporations of titanium (10 nm) and gold (100 nm) using a Temescal FC-2000 E-beam evaporator and lift-off technique yields interdigitated microband (55 μm × 1 μm × 60 nm) structures with gaps of 2 μm between the combs. A second metal evaporation and liftoff process yields the interconnection tracks, contact pads, and the gold counter electrode (90 μm × 7 mm). To prevent unwanted interactions along the connection tracks, silicon nitride, which acts as an insulating layer was deposited by plasma-enhanced chemical vapor deposition. Photolithography and dry etching were utilized to selectively open windows (45 μm × 100 μm) in the insulating SiN layer over the microband electrodes for electrolyte access. Openings were also created over the counter electrodes and the contact pads. Each device contains six IDEs (sensors) which are separated by a gap of 0.94 mm. Once the sensor fabrication is completed, a wafer is diced into 28 separate chip devices.

A custom-made holder cell was fabricated to allow measurement in small electrolyte volumes (≈50 μL to 5 mLs). The cell was constructed from an aluminum base and a Teflon lid. Spring-loaded probes (Coda Systems Ltd. PM4J Plain Radius Microprobes) were inserted into the lid in position above the peripheral contact pads, to permit electrical connection to external potentiostats. The cell was assembled with a Viton O-ring embedded in the lid to form a seal around the on-chip electrodes. Viton O-rings were chosen for their chemical resistance. The inner diameter of the O-ring was 7 mm with a cross section of 1.6 mm, to allow an opening large enough to expose all six sensors, counter, and reference electrodes on the device to the electrolyte. Typical volumes measured were 5 mL.

### Electrode Characterization

Each chip was inspected using optical microscopy to identify any obvious defects or faults. Prior to any electrochemical characterization, chips were cleaned by immersion in acetone, iso-propyl alcohol, and finally de-ionized water, each for a period of ten minutes. The chips were dried under a flow of nitrogen and placed in the chip holder. Electrochemical analysis was performed using an Autolab Bipotentiostat (MAC80150 with BA Module, Metrohm). Cyclic voltammograms (CV) were performed from 0 to 0.6 V at 50 mV/s in 1 mM ferrocene carboxylic acid (FCA, Sigma-Aldrich, 97%) dissolved in 10 mM phosphate buffered saline (PBS, Sigma-Aldrich). During these scans, the second interdigitated comb of electrodes was held at 0 V. All electrochemical measurements were recorded *versus* a SCE, in solutions at room temperature (21 °C).

### Platinum Plating on Protonator Electrode

Platinum plating was carried out on one comb of the IDE array to enhance the protonator oxygen evolution performance. This was achieved by biasing the comb at −0.5 V *versus* SCE in a commercial Platinum DNS bath (Johnson Matthey). Plating times from 6 to 12 s were investigated, but ultimately 8 s depositions were used. Scanning electron microscopy (SEM) and energy-dispersive X-ray spectroscopy (EDX) were also used to characterize the platinum deposition.

### Measurement of MCA by the Colorimetric Test

Colorimetric measurement of MCA concentration was performed on the stock solution and subsequent diluted working solutions to determine the concentration. This was undertaken as MCA can degrade over time, but more significantly, the hypochlorous acid content of bleach can also drop over time. The stock therefore may not always be 200 ppm, depending on when the bleach was purchased. A commercial test kit was employed to determine the concentrations of the MCA solutions. The Hach colorimeter and indophenol method was used for this quantification. 10 mL of the MCA stock solution was added to a clean sample vial. This was put into the colorimeter, and a blank measurement was taken. Monochlor F reagent was then added to the sample vial. This reagent was purchased as a preweighed sachet of powder, the entire contents of which were added to the sample. As the reagent reacted with the sample, the solution gradually became green with maximum coloration being achieved after a reaction time of 5 min. This was then put back into the colorimeter, and a measurement was taken. This method is only sensitive to concentrations between 0.5 and 5 ppm of MCA, so dilutions were required to determine the concentration of stock solutions.

### Electrochemical Analysis of Acidified MCA Solutions

Solutions of MCA in artificial drinking water (ADW) were acidified to pH 3 to determine the electrochemical behavior of DCA on the IDEs. ADW was prepared by dissolving 1 g of sodium bicarbonate, 0.0654 g of magnesium sulfate (Sigma-Aldrich, 99.5% anhydrous), 0.3414 g calcium sulfate dehydrate (honeywell, 99%), 0.007 g potassium phosphate dibasic (Fluka, 98%), potassium phosphate monobasic (Sigma-Aldrich, 99%), and 0.01 g sodium nitrate (Sigma-Aldrich, 99%) in 10 L of deionized water. Sulfuric acid (0.1 M H_2_SO_4_) was used to reduce the solution pH to the required value. The pH of each solution was confirmed with a pH meter (Hach). Electrochemical analysis of the acidified samples was performed using an Autolab potentiostat (MUX 101 with BA module) in a Faraday cage. The working electrode used was an IDE array with 1 μm wide electrodes separated by 2 μm gaps. The counter was an on-chip platinum electrode, and the reference was a SCE. CVs were performed from 1.2 to 0.2 V at 50 mV/s. Scans were also performed at intermediate pH values to determine the pH dependence of DCA formation.

### pH Control in MCA Solutions at μIDE Arrays

Electrochemical analysis of MCA solutions with pH control was carried out using an Autolab bipotentiostat in a Faraday cage. The same electrochemical parameters were used as for the acidified samples. The starting potential was reduced to 0.95 V for later work. The protonator electrode was held at 1.57 or 1.65 V depending on the matrix conditions. Working samples were made by diluting the MCA stock solution with ADW. Typical concentrations of 0.5 to 5 ppm MCA were used, with 10 ppm samples used to study the upper detection limit.

### Effects of Interferents and Matrix Composition on MCA Detection

Solutions of MCA with high alkalinity were prepared by the addition of sodium bicarbonate (0.84 g/500 mL ADW stock). Different MCA sample solutions were spiked with 1 ppm each of iron, copper, and phosphate [as iron(II) chloride, copper sulfate, and sodium phosphate monobasic] to determine their interferent’s effect on the MCA analysis.

## Results and Discussion

### Device Characterization

**Figure 2:**
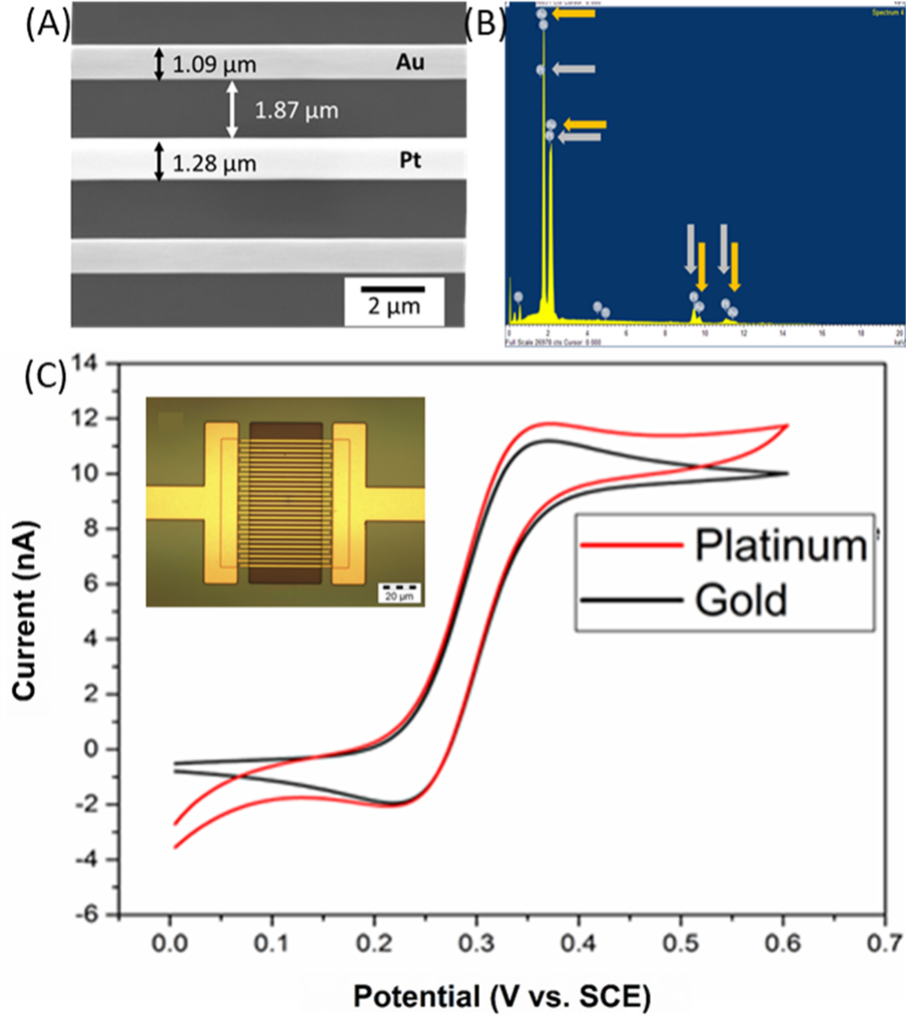
(A) SEM image of the IDE array showing one comb plated with platinum. (B) EDX spectra of the plated platinum comb of electrodes showing both platinum and gold peaks. (C) CV of a gold comb (black) and a platinum comb (red) of electrodes in a solution of 1 mmol/L FCA in 10 mmol/L PBS. CVs were performed from 0 to 0.6 V at 50 mV/s. The inset shows an image of the IDE taken at 100× magnification.

**Figure 3:**
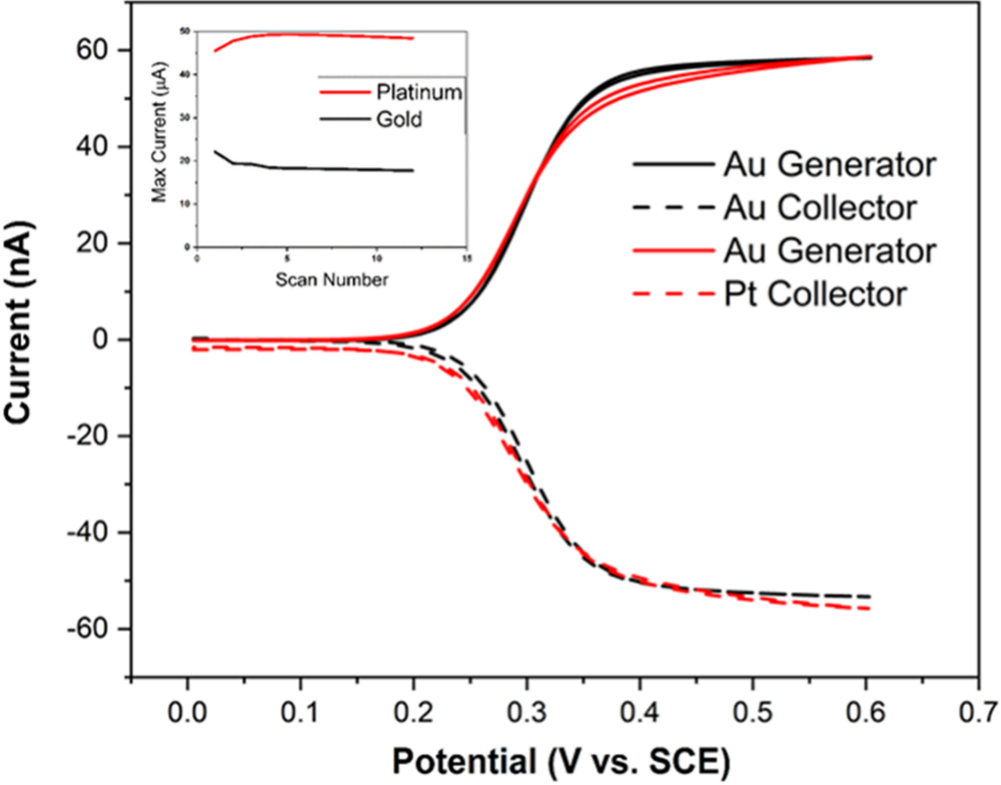
CVs of a gold–gold array (black) and a gold–platinum array (red) in generator collector mode. CVs were performed at the generator (solid line) in 1 mmol/L FCA in 10 mmol/L PBS from 0 to 0.6 V at 50 mV/s, while the collector (dashed line) was biased at 0 V. The inset shows the comparison of platinum (red) and gold (black) performance as a protonator over multiple scans.

Devices were fabricated with an interelectrode comb spacing of 2 μm. Each comb of the IDEs can be addressed separately, allowing for generator–collector-type sensing applications. In this work, platinum was plated onto one comb of the interdigitated array which was to be used as the protonator electrode. The platinum deposition was performed by biasing one comb of the gold interdigitated array at −0.5 V for 8 s. Figure 2A is a SEM image showing that platinum was successfully deposited solely onto one comb of electrodes, with the other comb being unaffected. The SEM image indicates that approximately a 100 nm thick deposit was achieved. The EDX shown in Figure 2B further shows the presence of platinum on the comb of electrodes, which is indicted by the platinum peaks highlighted with the gray arrows. Gold was also detected, as only a thin layer of platinum was deposited, through which the X-rays penetrated detecting the underlying gold. An enlarged version of Figure 2B is shown in the Supporting Information (SF2). Following SEM and EDX, the sensors were electrochemically characterized using FCA. Figure 2C shows a typical scan performed in single mode (*i.e.,* no bias at the collect comb of electrodes) at the gold comb and the platinum comb. The combs were swept from 0 to 0.6 V at 50 mV/s, oxidizing the FCA to FCA^+^. The CVs showed different behaviors for the gold and platinum combs of electrodes, further indicating successful deposition of platinum. The reduction event seen between 0 and 0.1 V for platinum is the onset of oxygen reduction, as platinum shows better catalytic activity toward this reaction than gold. In both cases, the CVs showed a peak associated with FCA oxidation at approximately 0.35 V. Steady-state behavior was not observed for the micron-scale electrodes, given that diffusional overlap resulted in the array behaving as one large electrode as opposed to multiple smaller electrodes. The platinum comb of electrodes showed a higher current for the FCA oxidation because of the increased surface area as a result of the plating procedure. Following characterization in single mode, the devices were characterized in generator–collector mode (*i.e.,* a bias imposed on the collector). In this case, the generator comb of electrodes was swept from 0 to 0.6 V at 50 mV/s, while the collector was biased at 0 V *versus* SCE. The generator comb oxidized the FCA to FCA+. The FCA+ species diffused across the gap to the collector electrode, which subsequently reduced it back to FCA. This is a phenomenon known as redox cycling and can be used to boost signals as described by Wahl *et al.*(40) The voltammograms shown in Figure 3 were typical of a fully working array. Figure 3 shows a comparison between a gold–gold IDE and a gold–platinum IDE. In both cases, gold was used as the generator electrode with the collector being either platinum or gold. It was found that steady-state behavior was achieved as the bias imposed at the collector electrodes prevented diffusional overlap. No significant change in behavior was observed between the gold–gold IDE and the gold–platinum IDE other than an increased collection efficiency. The collection efficiency is a measure of how much species generated at the generator is detected by the collector and is calculated by expressing the collector current as a percentage of the generator current. A collection efficiency of 91.1% was calculated for the gold–gold array, which indicated that a significant portion of the generated FCA^+^ was observed at the collector. The collection efficiency of the gold–platinum IDE, however, was calculated to be 95.7%. The reason for the increase in collection efficiency resulted from increasing the width of the electrode, which subsequently decreased the gap between the generator and the collector electrodes. The inset shown in Figure 3 is the current measured at gold and platinum electrodes when a potential, where oxygen evolution occurred, was applied in an ADW solution. Both electrodes were held at 1.65 V, and it was found that the current measured at platinum was significantly higher than gold over multiple scans, as shown in the inset in Figure 3. As the pH control method requires oxygen evolution to produce protons, the higher current measured at platinum indicates that it is a superior protonator material and was used in each of the pH control experiments.

### pH Dependence of DCA Formation

**Figure 4:**
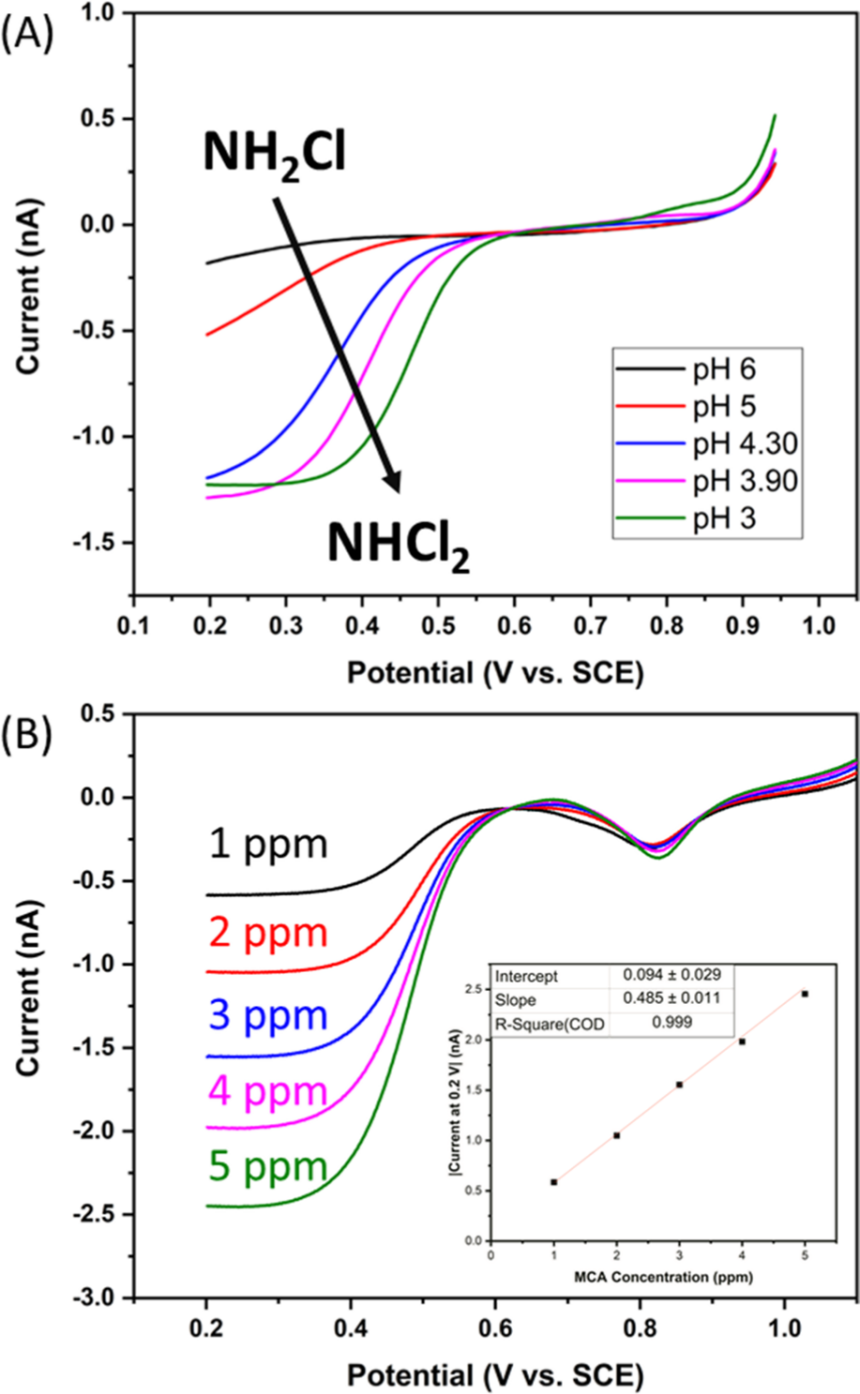
(A) Dependence of sample pH on the formation of DCA. (B) Electrochemical reduction of DCA at the gold comb of an IDE. CVs were performed in various concentrations of MCA in ADW that were subsequently adjusted to pH 3. LSVs were performed from 1.2 to 0.2 V at 50 mV/s. The inset shows the calibration plot with a slope of 0.485 and an *R*^2^ of 0.999, Error bars of triplicate measurements are within the data points.

To determine the pH value at which DCA is dominant and detectable by amperometry, LSVs were performed in samples with 2.5 ppm MCA in ADW that was adjusted to various pH values. This was performed by preparing samples of MCA in ADW at pH 8.5 and subsequently acidifying each sample with 0.1 M H_2_SO_4_ until the desired pH was achieved. Figure 4A shows the result of performing an LSV at the generator comb from 0.95 to 0.2 V at 50 mV/s with the collector left unbiased at each pH solution. It was found that at near neutral pH conditions, little to no DCA was detected. Full conversion to DCA was not observed until the sample pH was acidified to pH 3, which was indicated by the steady-state response. Subsequently, a calibration was performed in solutions of MCA acidified to pH 3. Sample concentrations between 1 and 5 ppm MCA were used for this calibration as this range encompasses the average values expected when using MCA as a disinfectant. Each sample was acidified to pH 3 to ensure the complete conversion of MCA to DCA. LSVs were performed at the generator comb in each solution from 1.2 to 0.2 V, while the collector comb was again left unbiased. These parameters were chosen as this allows a surface gold oxide to form. The reduction potential can be used as an indicator of sample pH, which was a useful internal probe to determine if the pH control method is sufficient. Figure 4B shows the LSVs for each concentration. A steady-state response was observed for the reduction of DCA, and the inset shows the corresponding calibration plot. A sensitivity to DCA of 0.485 nA/ppm was achieved with an *R*^2^ of 0.999, showing that the reduction current was directly related to the MCA concentration.

### Analysis of MCA Samples by Conversion to DCA Using *In Situ* pH Control

**Figure 5:**
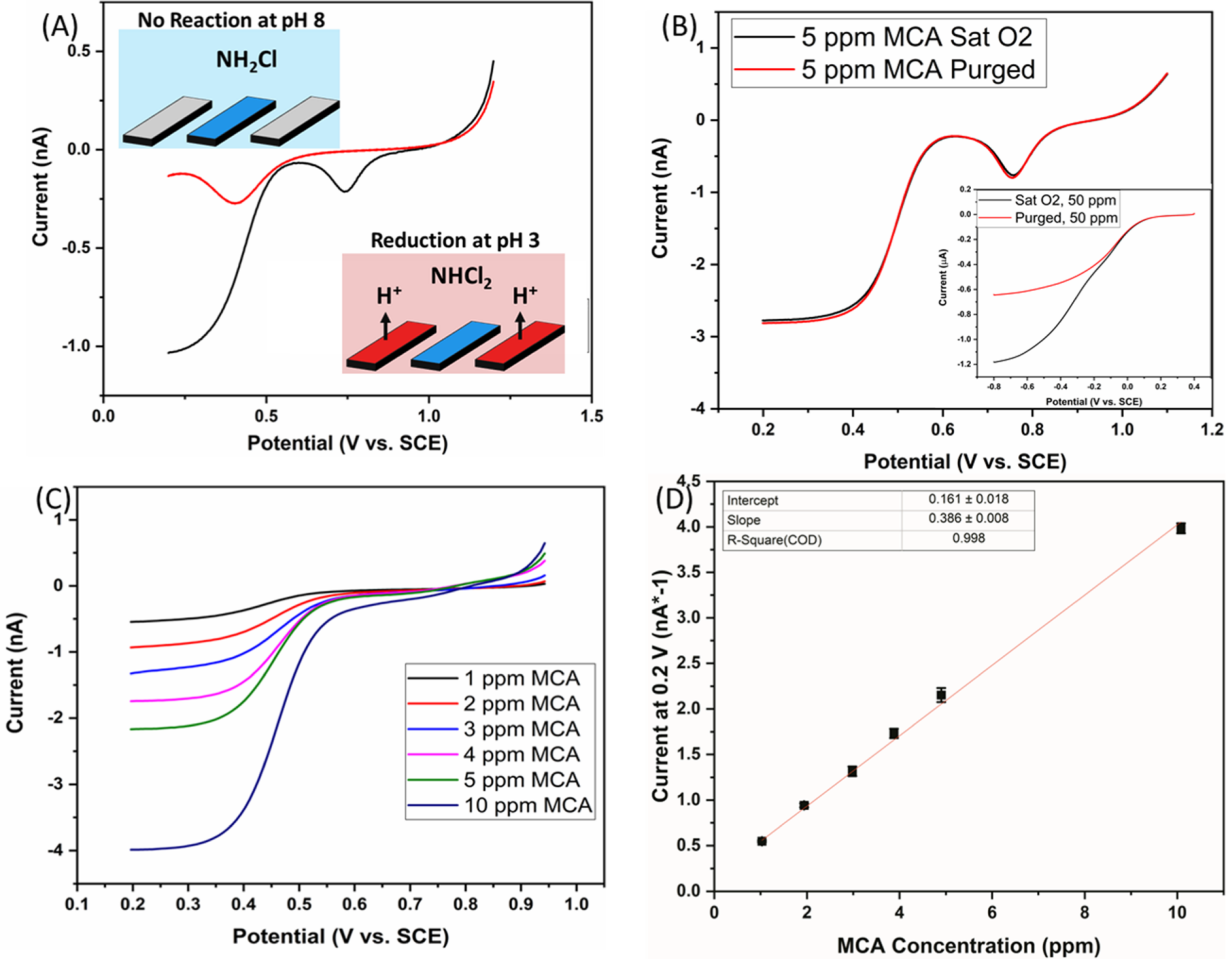
Comparison of LSVs in 2 ppm MCA with *in situ* pH control off (red) and on (black). CVs were performed at a gold comb of an IDE from 1.2 to 0.2 V at 50 mV/s with the platinum protonator comb biased at 1.57 V. The insets show a schematic of the electrode environment. (B) Comparison of LSVs in 5 ppm MCA samples with the pH control method applied at high and low concentrations of oxygen. The inset shows LSVs performed at a commercial microdisc electrode in 50 ppm samples of MCA at high and low oxygen concentrations from 0.4 to −0.8 V at 50 mV/s. (C) LSVs in MCA samples from 1 to 10 ppm with the applied pH control method. LSVs were performed at a gold comb of electrodes from 0.95 to 0.2 V at 50 mV/s with the platinum protonator biased at 1.57 V. (D) Calibration plot for the scans performed in (C).

Having established that the MCA samples were converted to DCA at pH 3, and that this was quantifiable with the developed sensors, tests were performed using the proposed *in situ* pH control method. Figure 5A shows the initial LSVs performed in a 2 ppm sample of MCA. The voltammogram shows a comparison between the situation when pH control was “off” and subsequently when *in situ* pH control was “on”. The LSVs involved scanning the sensor comb of electrodes from 1.2 to 0.2 V at 50 mV/s, while the protonator combs were unbiased (pH control off) or biased at 1.57 V (pH control on). When the pH control technique was not applied, the solution contained only the added MCA, and no activity is seen in this potential window. The gold oxide reduction peak is seen at 0.3 V, which was indicative of the prepared pH 8.5 solution. When the pH control method was applied, acidification of the solution in the vicinity of the sensing electrodes drives it to pH 3 and MCA converted to DCA. In this case, a reduction event was observed which was similar to the equivalent scan performed in the chemically adjusted scans, as shown in Figure 4B. The gold oxide reduction peak is shifted to a potential of 0.75 V indicating that the local environment was pH 3.4, thus more acidic than the bulk conditions. Figure 5B shows a comparison of LSVs performed in samples saturated with oxygen and those wherein the oxygen had been purged entirely by nitrogen degassing. It shows that oxygen had no impact on the amperometric detection of DCA, and oxygen interference is eliminated. The inset shows a scan performed using a commercial microdisc electrode in a 50 ppm solution of MCA. In this case, the electrode was swept from 0.4 to −0.8 V at 50 mV/s, which is the potential window wherein we can observe MCA reduction. A comparison between saturated and purged samples is shown, and it is clear that oxygen has a significant impact on MCA detection. It was found that a 0.6 μA difference was attributed to the presence of oxygen, effectively doubling the signal related to the 50 ppm sample. For a 5 ppm sample, this would correspond to a near ten-fold increase in the signal. Variability of oxygen concentration therefore creates significant uncertainty in the detection of MCA. The pH control method was applied to various concentrations of MCA in ADW, as shown in Figure 5C. In these LSVs, the sensor comb was swept from 0.95 to 0.2 V at 50 mV/s, while the protonator comb was biased at 1.57 V. As the pH control parameters were established, the generation of the oxide was no longer necessary. It had also been found that the presence of chlorine was causing dissolution of gold at 1.2 V; therefore, the lifetime of the sensor was improved by narrowing the potential window. For each concentration, the behavior was similar to that observed in the chemically adjusted pH samples. The typical range for MCA is 1 to 5 ppm; however, this sensor was tested up to 10 ppm MCA. This was undertaken as MCA can be formed if an excess of hypochlorite is present. In such a case, extreme MCA concentrations may be observed. The sensor was tested in such conditions to determine if the high concentrations could be effectively converted to DCA. Figure 5D shows the calibration plot for the scans, as shown in Figure 5C. Again, a good linearity was observed with an *R*^2^ value of 0.998. The measured sensitivity was found to be 0.385 nA/ppm, which was lower than the scans performed in the chemically adjusted samples. This has been attributed to the lack of gold oxide formation and subsequent reduction, which did not occur in these scans. The formation of gold oxide and subsequent reduction directly before the sensing measurement ensured a reproducible electrode surface. This process can remove adsorbed species that may influence the sensitivity. As this was not performed in the pH control measurements to preserve the electrode lifetime, a slightly lower sensitivity was measured. This was confirmed by performing scans with the pH control method and the formation of a gold oxide, wherein the calibration plot indicates a sensitivity comparable to the pH adjusted samples. This data is shown in the Supporting Information (SF3). A limit of detection was calculated for this method using the standard error of the estimate approach (SEq1).^41^ However, rather than using a blank sample, seven replicates of a sample with 0.5 ppm MCA were used resulting in a calculated limit of detection of 0.03 ppm. From this calibration, a series of samples containing 2 ppm MCA were compared across five sensors. The concentrations of MCA present were calculated using the calibration plot and simultaneously by using the standard colorimetric method which showed for this sensing approach an average variation of 3.08% with a maximum deviation of 4.62%, the data for which is shown in the Supporting Information (SF4).

### Determination of the Effects of Matrix Composition and Common Interferents

**Figure 6:**
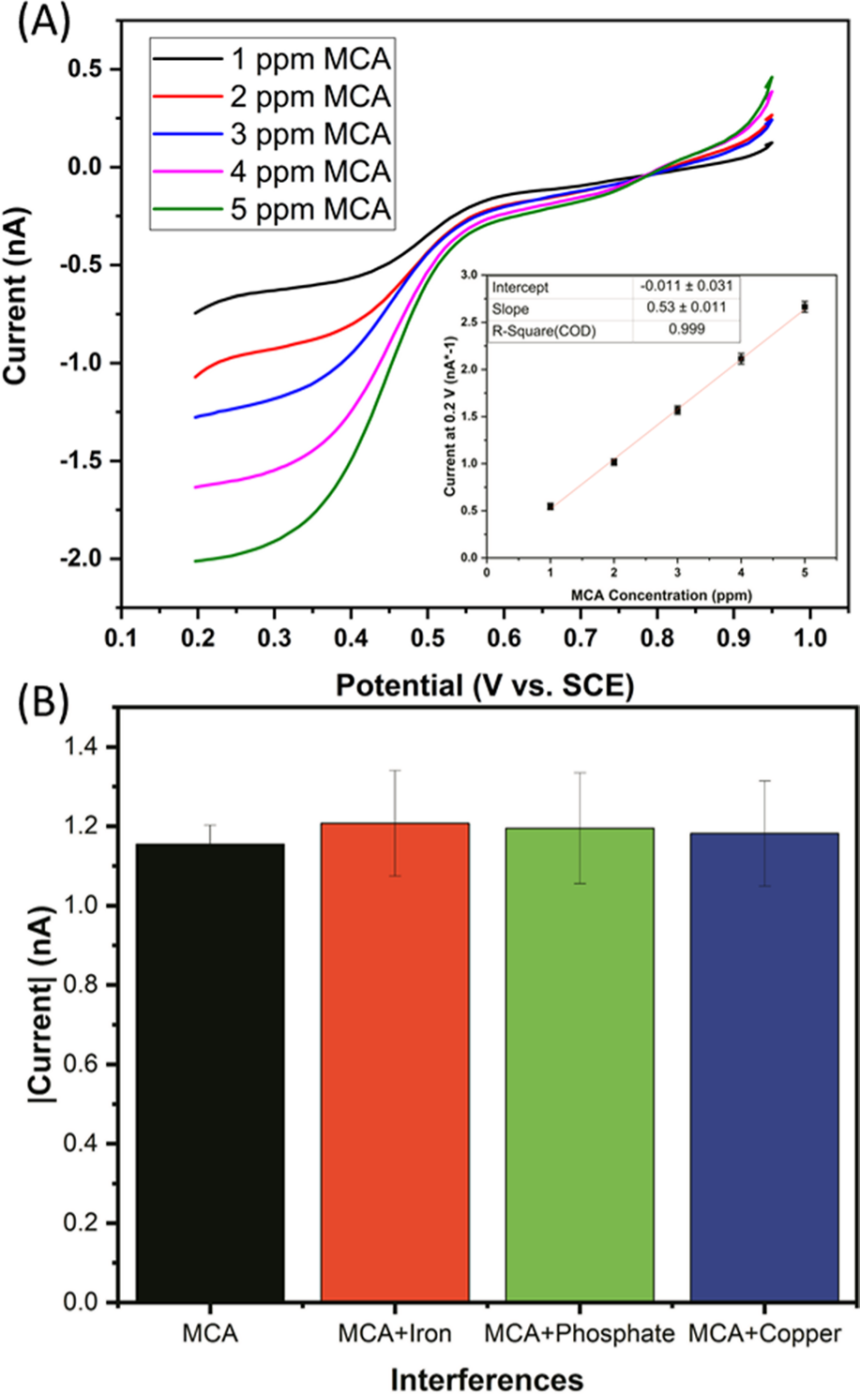
(A) LSVs in various concentrations of MCA in high alkalinity ADW. LSVs were performed at a gold comb of electrodes from 0.95 to 0.2 V at 50 mV/s with the platinum protonator biased at 1.65 V. The inset shows the calibration plot with a slope of 0.363 and an *R*^2^ of 0.998. (B) Comparison of a 2.5 ppm MCA sample to equivalent samples spiked with 1 ppm of iron, phosphate, and copper.

As water systems can be quite complex, oxygen is not the only anticipated interferent. Therefore, tests were performed to determine the viability of the sensor in the presence of additional interference. The sample alkalinity was of the most concern for this testing method as this can increase the buffering capacity of water. As the proposed method relies on changing pH, high buffering capacity can add difficulty to this approach. The alkalinity of a water sample can be quite variable and typically is not a huge health concern, so high alkalinities can be common in some water systems. High alkalinity is expected to have approximately 500 ppm of carbonates or bicarbonates with 1000 ppm being regarded as very high. For this reason, tests were performed in samples of ADW with the addition of 1000 ppm sodium bicarbonate as a worst-case scenario. Figure 6A shows the LSVs performed using the pH control method in high alkalinity samples. The major difference in parameters was an increase in the protonator potential from 1.57 to 1.65 V. This was required to achieve the desired pH control. The LSV performed at the sensor comb was kept at 0.95 to 0.2 V at 50 mV/s. The inset shows a calibration plot with an *R*^2^ of 0.998, indicating a good linearity. Interestingly, the sensitivity measured was 0.363 nA/ppm. This is only slightly lower that the sensitivity measured in the low alkalinity samples (0.386 nA/ppm) which indicates that the sensor performance is not significantly affected by the alkalinity. The limit of detection in this case was also identical at 0.03 ppm using the same approach as for the low alkalinity samples. The increase to 1.65 V was employed as the measured current at the protonator was lower in the high alkalinity samples. If the protonator was biased with a current rather than a potential, this potential change would not be required. However, due to the limitations of the potentiostat, a current could not be applied at one comb of electrodes, while a potential sweep was performed at the other without the presence of a second counter electrode. As with the low alkalinity samples, a series of 2 ppm samples were made in the high alkalinity ADW and tested across five sensors. In this case, an average variability of 2.47% was observed with a maximum variation of 6.3%. Figure 6B shows the result of a series of LSVs performed in a high alkalinity sample of 2.5 ppm MCA in which 1 ppm of each interferent was added. The concentrations of the interferent added represent again a worst-case scenario for each species. This concentration represents a typical upper level for copper and a significantly high level for phosphate. For iron, higher concentrations may be found; however, at 1 ppm, iron is observable without additional equipment, so its presence is obvious. No obvious events were observed for each species, as shown in the Supporting Information (SF6); an increased current was measured in the presence of each species. A 4.6% increase over the expected current for a 2.5 ppm MCA sample was observed for iron. For phosphate and copper, the increase was found to be 3.5 and 2.4%, respectively.

## Conclusions

In this paper, we have demonstrated that reliable, reagent-free detection of MCA was achieved by using an *in situ* pH control method. This method eliminates oxygen as an interfering species, which is one of the key difficulties associated with amperometric detection of MCA. The developed sensor was calibrated between 0 and 5 ppm MCA, which is the typical range expected in potable water, showing both accuracy and precision in measurements, with deviations of less than 6% and a detection limit calculated at 0.03 ppm MCA. The upper extreme was tested using a 10 ppm MCA sample, which was quantified with no loss of sensor performance. These ranges of concentrations far surpass the anticipated limits of potable water, indicating that the developed sensor is more than suitable for real-world applications. The greatest anticipated challenge facing the use of pH control in real water samples is the inherent buffering capacity of water because of its alkalinity. In this work, we have evaluated the detection method at a potential worst-case scenario for alkalinity, wherein the concentration of carbonates was 1000 ppm and found that the sensor performance was not impacted. The presence of common interferents in water had no impact on the detection method and the MCA was still quantifiable in the presence of such compounds.

## Supporting Information Available

pH dependence on chloramine distribution, enhanced EDX data, MCA scans with oxide formation, LOD calculations, data for accuracy of test measurements, and LSV’s for interference tests (PDF)

## Notes

A preprint of this manuscript was made available on ChemRxiv. Seymour, Ian; O’Sullivan, Benjamin; Lovera, Pierre; O’Riordan, Alan; Rohan, James: Elimination of Oxygen Interference in the Electrochemical Detection of Monochloramine Using In Situ pH Control at Interdigitated Electrodes. (2020) ChemRxiv. Preprint. https://doi.org/10.26434/chemrxiv.12743720.v1 (Accessed 02/02/2021).
