# Supplementary material for: Elimination of Oxygen Interference in the Electrochemical Detection of Monochloramine, Using In Situ pH Control at Interdigitated Electrodes
Source: ACS Sens. 2021 Feb 22;6(3):1030–8. doi: 10.1021/acssensors.0c02264 (PMC8815066; doi:10.1021/acssensors.0c02264)
Supplement: Supplementary file 1 — se0c02264_si_001.pdf [file se0c02264_si_001.pdf]

Supporting Material – Elimination of Oxygen Interference in the Electrochemical Detection of Monochloramine, using in-situ pH Control at Interdigitated Electrodes

Ian Seymour, Benjamin O'Sullivan, Pierre Lovera, Alan O'Riordan* and James F. Rohan

Tyndall National Institute, Cork, Ireland.
E-mail: alan.oriordan@tyndall.ie

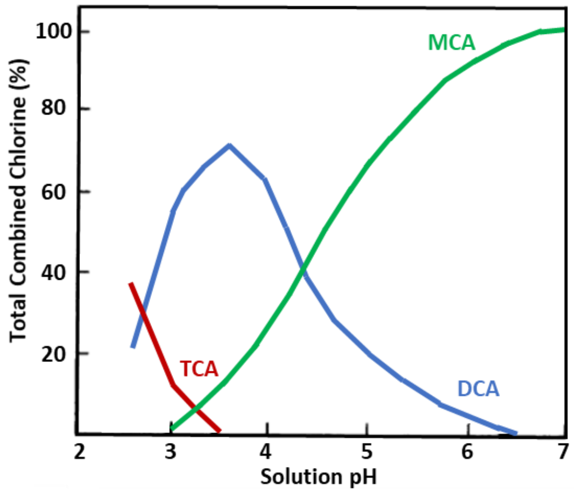

Supporting Figure 1: Combined chlorine as a function of pH. Sample exists as monochloramine (green), dichloramine (blue) or trichloramine (red) depending on the sample acidity.

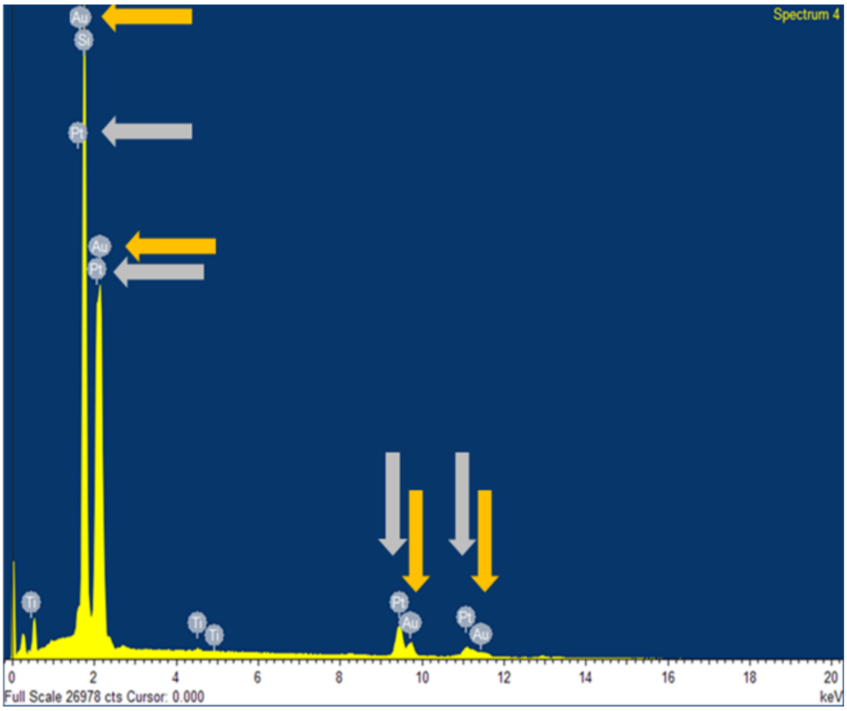

Supporting Figure 2: Enlarged image of the EDX data for the platinum deposition

Supporting figure 3 shows the LSVs and subsequently calibration of scans performed in various concentrations of MCA in ADW at pH 8.5. The major difference for these scans is that the gold oxide peak was allowed to form and subsequently reduce prior to the reduction of DCA. This resulted in a cleaner gold surface which has been attributed to the increased sensitivity shown in the calibration plot (0.513 nA/ppm)

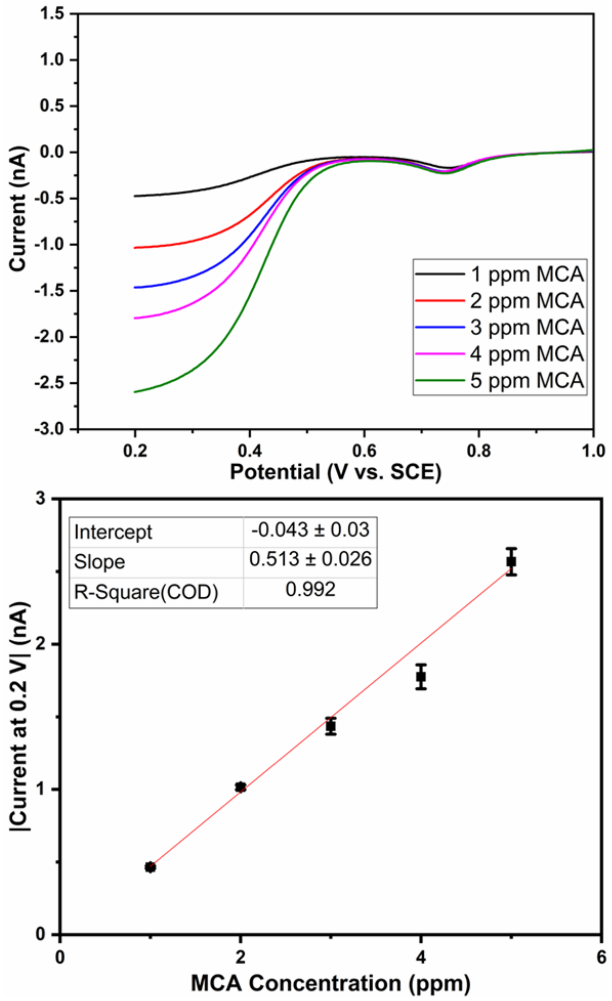

Supporting Figure 3: **TOP:** LSVs performed in various concentrations of MCA using the pH control method. The sensor electrode was swept from 1.2 V to 0.2 V at 50 mV/s while the protonator was biased at 1.57 V. **BOTTOM:** The calibration of the current at 0.2 V for each concentration of MCA. Error was calculated from the standard deviation of 3 replicate scans

The limit of detection (LOD) was calculated using the standard error of estimate equation (SEq 1)

$$LOD = 3.3\ x\ \frac{\sigma_s}{m} \qquad \textbf{SEq.1}$$

Where σ_s is the standard deviation of a low concentration sample and m is the slope of the calibration plot.

Measured MCA *ppm*	Predicted MCA *ppm*	Variation *%*	Measured Current *nA*	Predicted Current *nA*	Variation *%*
2.09	2.14	2.44	-0.96	-0.94	2.38
1.97	2.05	3.66	-0.92	-0.89	3.53
1.97	2.04	3.18	-0.92	-0.89	3.08
1.92	1.89	1.82	-0.85	-0.87	1.77
2.01	1.92	4.61	-0.87	-0.91	4.62

Supporting Figure 4: Comparison of the colorimetric concentration measurements and the electrochemical concentration measurements for five MCA samples on separate IDE devices. These measurements were obtained using the pH control method in ADW samples.

Measured MCA *ppm*	Predicted MCA *ppm*	Variation *%*	Measured Current *nA*	Predicted Current *nA*	Variation *%*
1.97	2.10	6.60	-0.96	-0.91	6.30
1.98	2.03	2.48	-0.92	-0.92	2.07
1.95	2.01	3.28	-0.92	-0.90	2.72
1.96	1.95	0.60	-0.85	-0.91	0.52
1.96	1.94	0.86	-0.87	-0.91	0.74

Supporting Figure 5: Comparison of the colorimetric concentration measurements and the electrochemical concentration measurements for five MCA samples on separate IDE devices. These measurements were obtained using the pH control method in high alkalinity ADW samples.

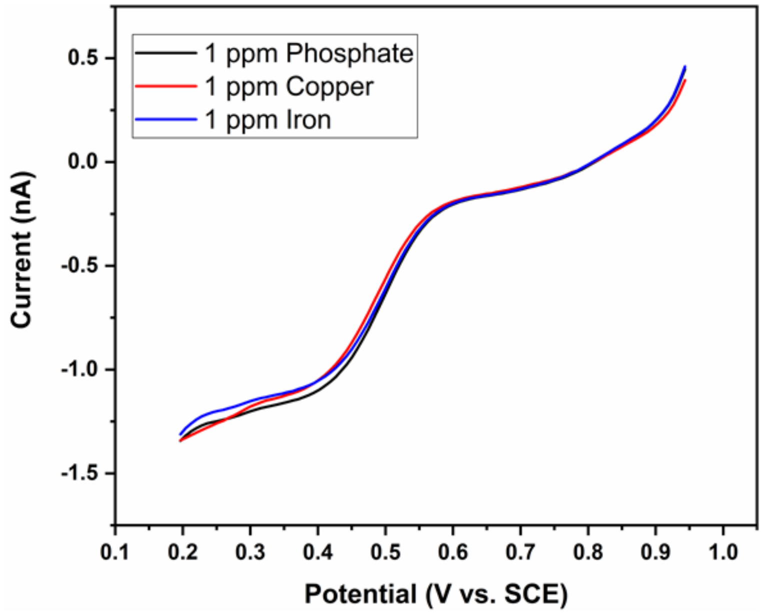

Supporting Figure 6: LSVs of 2.5 ppm MCA in the presence of various interferents. The sensor was swept from 0.95 V to 0.2 V at 50 mV/s while the protonator was biased at 1.65 V
